# Germline RAD51B variants confer susceptibility to breast and ovarian cancers deficient in homologous recombination

**DOI:** 10.1038/s41523-021-00339-0

**Published:** 2021-10-11

**Authors:** Jeremy Setton, Pier Selenica, Semanti Mukherjee, Rachna Shah, Isabella Pecorari, Biko McMillan, Isaac X. Pei, Yelena Kemel, Ozge Ceyhan-Birsoy, Margaret Sheehan, Kaitlyn Tkachuk, David N. Brown, Liying Zhang, Karen Cadoo, Simon Powell, Britta Weigelt, Mark Robson, Nadeem Riaz, Kenneth Offit, Jorge S. Reis-Filho, Diana Mandelker

**Affiliations:** 1 Department of Radiation Oncology, Memorial Sloan Kettering Cancer Center, New York, NY 10065 USA; 2 Department of Pathology, Memorial Sloan Kettering Cancer Center, New York, NY 10065 USA; 3 GROW School for Ontology and Developmental Biology, University of Maastricht, Maastricht, The Netherlands; 4 Department of Medicine, Memorial Sloan Kettering Cancer Center, New York, NY 10065 USA; 5 Niehaus Center of Inherited Cancer Genomics, Memorial Sloan Kettering Cancer Center, New York, NY 10065 USA; 6 Molecular Biology Program, Sloan Kettering Institute, New York, NY 10065 USA

**Keywords:** Cancer genetics, Cancer genetics

## Abstract

Pathogenic germline mutations in the RAD51 paralog genes *RAD51C* and *RAD51D*, are known to confer susceptibility to ovarian and triple-negative breast cancer. Here, we investigated whether germline loss-of-function variants affecting another RAD51 paralog gene, *RAD51B*, are also associated with breast and ovarian cancer. Among 3422 consecutively accrued breast and ovarian cancer patients consented to tumor/germline sequencing, the observed carrier frequency of loss-of-function germline *RAD51B* variants was significantly higher than control cases from the gnomAD population database (0.26% vs 0.09%), with an odds ratio of 2.69 (95% CI: 1.4–5.3). Furthermore, we demonstrate that tumors harboring biallelic *RAD51B* alteration are deficient in homologous recombination DNA repair deficiency (HRD), as evidenced by analysis of sequencing data and in vitro functional assays. Our findings suggest that *RAD51B* should be considered as an addition to clinical germline testing panels for breast and ovarian cancer susceptibility.

## Introduction

Approximately 5–10% of breast cancers and 20–25% of ovarian cancers are hereditary in nature^1^. Pathogenic germline variants in homologous recombination (HR)-related genes, including *BRCA1* (MIM: 113705), *BRCA2* (MIM: 600185), *BRIP1* (MIM: 605882), *PALB2* (MIM: 610355), *RAD51C* (MIM: 602774), and *RAD51D* (MIM: 602954), confer an increased risk of breast and/or ovarian cancers^2–7^. For female carriers of *RAD51C* or *RAD51D* pathogenic germline variants, the National Comprehensive Cancer Network recommends considering risk-reducing salpingo-oophorectomy to minimize the risk of ovarian cancer^8^. The HR deficiency (HRD) characteristic of tumors that arise in individuals harboring germline mutations in HR-related genes can be exploited therapeutically with poly(ADP-ribose) polymerase (PARP) inhibitors and platinum compounds^9,10^.

*RAD51B* (MIM: 602948) encodes one of five classical RAD51 paralogs (*RAD51B*, *RAD51C*, *RAD51D*, *XRCC2* [MIM: 600375], and *XRCC3* [MIM: 600675]) known to be required for HR and maintenance of genomic stability^11^. Structurally related to RAD51, the paralogs are not thought to have a direct role in homology recognition, but act as accessory factors required for proper function of the core RAD51 recombinase^12,13^. Whilst *RAD51C* and *RAD51D* are now established cancer predisposition genes, loss-of-function germline variants in *RAD51B* have only been reported in individual cases of breast and ovarian cancer^14–16^. Here, we sought to determine whether germline loss-of-function variants in *RAD51B* confer an increased risk to these cancers, and whether the resulting tumors harbor a therapeutically targetable HRD phenotype.

## Results

### Germline RAD51B variants and cancer predisposition

To investigate the potential contribution of *RAD51B* loss-of-function germline variants to cancer predisposition, we analyzed a cohort of 18,087 individuals with cancer whose tumor and blood samples were characterized using MSK-IMPACT. Among the cancers represented in this cohort were 2265 breast and 1157 ovarian cancers. Overall, 13 (0.07%) cases were found to harbor a *RAD51B* loss-of-function germline variant, a frequency similar to that described in the gnomAD database (0.09%). Notably, 9/10 of the female carriers of a *RAD51B* loss-of-function germline variant had a diagnosis of breast or ovarian cancer (5/2265 and 4/1157), for an odds ratio for breast and ovarian cancer susceptibility of 2.69 (95% CI: 1.4–5.3, *p* = 0.004; Table 1). Though segregation data were not available, 8/13 of the *RAD51B* loss-of-function germline variant carriers had a first or second degree relative with breast or ovarian cancer (Table 1; Supplementary Fig. 3).

**Table 1:** Germline *RAD51B* pathogenic variants in MSK vs. gnomAD cohorts.

	MSK cohort			gnomAD cohort			Comparative Statistics		
	Individuals with *RAD51B* truncating variant (*n*)	Total individuals (*n*)	Carrier frequency (%)	Individuals with *RAD51B* truncating variant (*n*)	Total individuals (*n*)	Carrier frequency (%)	OR	95%CI	*P* value
Pan-cancer	13	18,087	0.07%	136	138,632	0.10%	0.73	0.4–1.3	0.28
Breast and ovarian cancer	9	3422	0.26%	136	138,632	0.10%	2.69	1.4–5.3	0.004

*MSK* Memorial Sloan Kettering, *gnomAD* The Genome Aggregation Database, *OR* odds ratio, *CI* confidence interval, *NP* not performed.

### Genomic landscape of RAD51B-deficient tumors

**Fig. 1:**
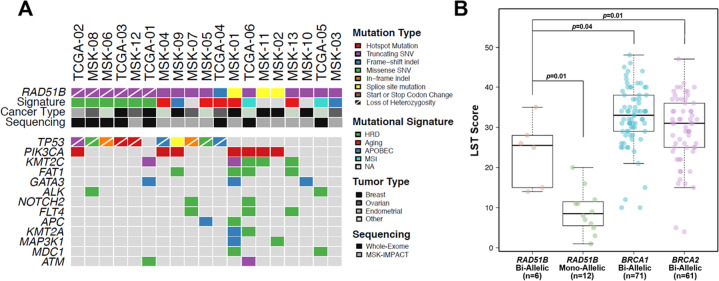
Genomic landscape of *RAD51B*-associated cancers. **A** Recurrent (present in ≥2 samples) nonsynonymous somatic mutations identified in 18 tumors from patients with germline *RAD51B* mutations using targeted massively parallel sequencing (MSK-IMPACT; *n* = 8) or whole-exome sequencing (WES; *n* = 8). Phenobar provides information on *RAD51B* germline mutations, dominant mutational signatures and cancer type. Loss of heterozygosity (LOH) of the *RAD51B* wild-type allele is displayed by a white diagonal line. **B** Large-scale transition (LST) scores in biallelic *RAD51B*-associated cancers, monoallelic *RAD51B*-associated cancers, biallelic *BRCA1*-associated cancers and biallelic *BRCA2*-associated cancers from TCGA.

**Fig. 2:**
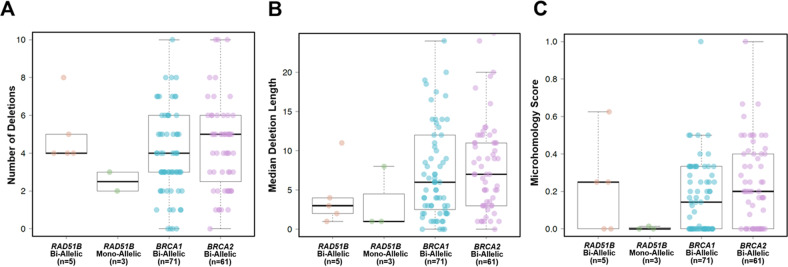
Analysis of genomic deletions in *RAD51B*-associated cancers. **A** Number of deletions in biallelic *RAD51B*-associated cancers, monoallelic *RAD51B*-associated cancers, biallelic *BRCA1*-associated cancers and biallelic *BRCA2*-associated cancers. Note one mon-allelic *RAD51B* was removed from the plot due to a large number of indels (142) driven by microsatellite instability. **B** Median deletion length in biallelic *RAD51B*-associated cancers, monoallelic *RAD51B*-associated cancers, biallelic *BRCA1*-associated cancers and biallelic BRCA2-associated cancers. **C** Deletion microhomology scores in biallelic *RAD51B*-associated cancers, monoallelic *RAD51B*-associated cancers, biallelic *BRCA1*-associated cancers and biallelic *BRCA2*-associated cancers. A deletion was classified as having microhomology when deletion length was ≥4 bases with homology ≥3 bases. Microhomology scores are calculated by dividing the total number of deletions with microhomology by the total number of deletions.

Given the observed association of *RAD51B* germline truncating variants with breast and ovarian cancer, we then sought to determine the phenotype and repertoire of somatic genetic alterations in cancers arising in *RAD51B* carriers. Among breast cancers, 4/5 were ER and PR positive and HER2 negative, while the remaining case was ER and PR negative and HER2 positive (Table 2). The average age of breast cancer diagnosis in *RAD51B* carriers was 49 (range 30–61), and ovarian cancer diagnosis was 67.3 (range 58–79). Six additional cases were identified in The Cancer Genome Atlas (TCGA), of which three had biallelic alterations of *RAD51B* (Supplementary Table 1). The six cases with biallelic *RAD51B* loss-of-function germline variants (germline variant plus loss-of-heterozygosity (LOH) of the wild-type (WT) allele) consistently displayed genomic features of HRD. All 5 cases with biallelic *RAD51B* mutation (germline plus LOH) for whom WES was performed harbored dominant signature 3 (Fig. 1A) and high large-scale state transition (LST) scores (Fig. 1B), indicative of defective HR in the tumor^17^. Tumors harboring biallelic inactivation of *RAD51B* displayed a small numerical increase in the number of deletions, deletion lengths, and microhomology scores, although this did not reach statistical significance (Fig. 2). Five of six cases with biallelic *RAD51B* inactivation also harbored biallelic somatic variants in *TP53*, which are known to be strongly selected for in BRCA-associated HRD cancers^18^. As LOH events themselves are copy number alterations (CNAs) that could contribute to LST signal, we compared biallelic *RAD51B* loss-of-function cases with WT *RAD51B* cases harboring LOH, and found those harboring a loss-of-function *RAD51B* allele to have significantly higher LST scores on average (*p* = 0.02), providing further supportive evidence that *RAD51B* loss leads to genomic scarring characteristic of defective HR, albeit at intermediate levels compared to *BRCA1* or *BRCA2* loss (Supplementary Fig. 1). Loss of heterozygosity of the WT allele was also observed at a significantly higher rate among tumors harboring a *RAD51B* loss-of-function germline variant (6/18 cases) vs. those with a variant of unknown significance (VUS; 6/59 cases, *p* = 0.02), suggesting selection for biallelic loss of *RAD51B* in tumors.

**Table 2:** Clinical characteristics of germline *RAD51B* pathogenic variant carriers in MSK cohort.

ID	Sex	Tumor type	*RAD51B* variant	*RAD51B* mutation type	Mean allele frequency (MAF) in gnomAD	Ethnicity-specific MAF in gnomAD	Age	Hormone receptor status	1st or 2nd degree relative with breast or ovarian cancer	Ethnicity	Notes
MSK_01	Female	Breast Invasive Ductal Carcinoma	c.85-1 G > C	Essential splice site SNV	1/246274 (0.0004)	0/33148 (0%)	61	ER+, PR+, HER2−	Yes, breast cancer	Hispanic/Latino	Bilateral breast cancer
MSK_02	Female	Breast Invasive Ductal Carcinoma	c.1036 + 2 T > C	Essential splice site SNV	Absent	Absent	41	ER+, PR+, HER2−	No	Turkish	Multifocal breast cancer
MSK_03	Female	High-Grade Serous Ovarian Cancer	c.37 C > T(p.Gln13*)	Truncating SNV	2/251326 (0.0008%)	2/30586 (0.007%)	64	NP	Yes, breast cancer	Hispanic/Latino	Synchronous endometrial cancer
MSK_04	Male	Unclassified Renal Cell Carcinoma	NM_002877.6(RAD51B): c.139 C > T(p.Arg47*)	Truncating SNV	38/282688 (0.01%)	12/129140 (0.009%)	51	NP	No	Caucasian	Synchronous prostate cancer
MSK_05	Male	High-Grade Neuroendocrine Carcinoma of the Colon/Rectum	NM_002877.6(RAD51B): c.139 C > T(p.Arg47*)	Truncating SNV	38/282688 (0.01%)	2/35374 (0.006%)	44	NP	No	Hispanic/Latino	
MSK_06	Male	Adenocarcinoma of the Gastroesophageal Junction	NM_002877.6(RAD51B): c.139 C > T(p.Arg47*)	Truncating SNV	38/282688 (0.01%)	2/35374 (0.006%)	31	NP	Yes, breast and ovarian cancer	Hispanic/Latino	
MSK_07	Female	High-Grade Serous Ovarian Cancer	NM_002877.6(RAD51B): c.139 C > T(p.Arg47*)	Truncating SNV	38/282688 (0.01%)	12/129140 (0.009%)	58	NP	Yes, breast cancer	Caucasian	
MSK_08	Female	High-Grade Serous Ovarian Cancer	NM_002877.6(RAD51B): c.139 C > T(p.Arg47*)	Truncating SNV	38/282688 (0.01%)	12/129140 (0.009%)	68	NP	Yes, breast cancer	Caucasian	
MSK_09	Female	Breast Invasive Ductal Carcinoma	c.204 T > A(p.Tyr68*)	Truncating SNV	Absent	Absent	48	ER−, PR−, HER2+	Yes, breast cancer	Hispanic/Latino	
MSK_10	Female	Breast Invasive Ductal Carcinoma	c.952 A > T(p.Arg318*)	Truncating SNV	1/31408 (0.003%)	1/15434 (0.006%)	30	ER+, PR+, HER2−	No	Caucasian	Also a *BLM* p.Ser595* carrier
MSK_11	Female	Breast Invasive Ductal Carcinoma	c.85-2delA	Essential splice site Indel	Absent	Absent	43	ER+, PR+, HER2−	No	Turkish	
MSK_12	Female	High-Grade Serous Ovarian Cancer	NM_002877.6(RAD51B): c.139 C > T(p.Arg47*)	Truncating SNV	38/282688 (0.01%)	2/35374 (0.006%)	79	NP	Yes, breast cancer	Hispanic/Latino	
MSK_13	Female	Melanoma	NM_002877.6(RAD51B): c.139 C > T(p.Arg47*)	Truncating SNV	38/282688 (0.01%)	12/129140 (0.009%)	67	NP	Yes, breast cancer	Caucasian	

*gnomAD* The Genome Aggregation Database, *ER* estrogen receptor, *PR* progesterone receptor, *HER2* human epidermal growth factor receptor 2, *NP* not performed.

### Functional impact of RAD51B deficiency

**Fig. 3:**
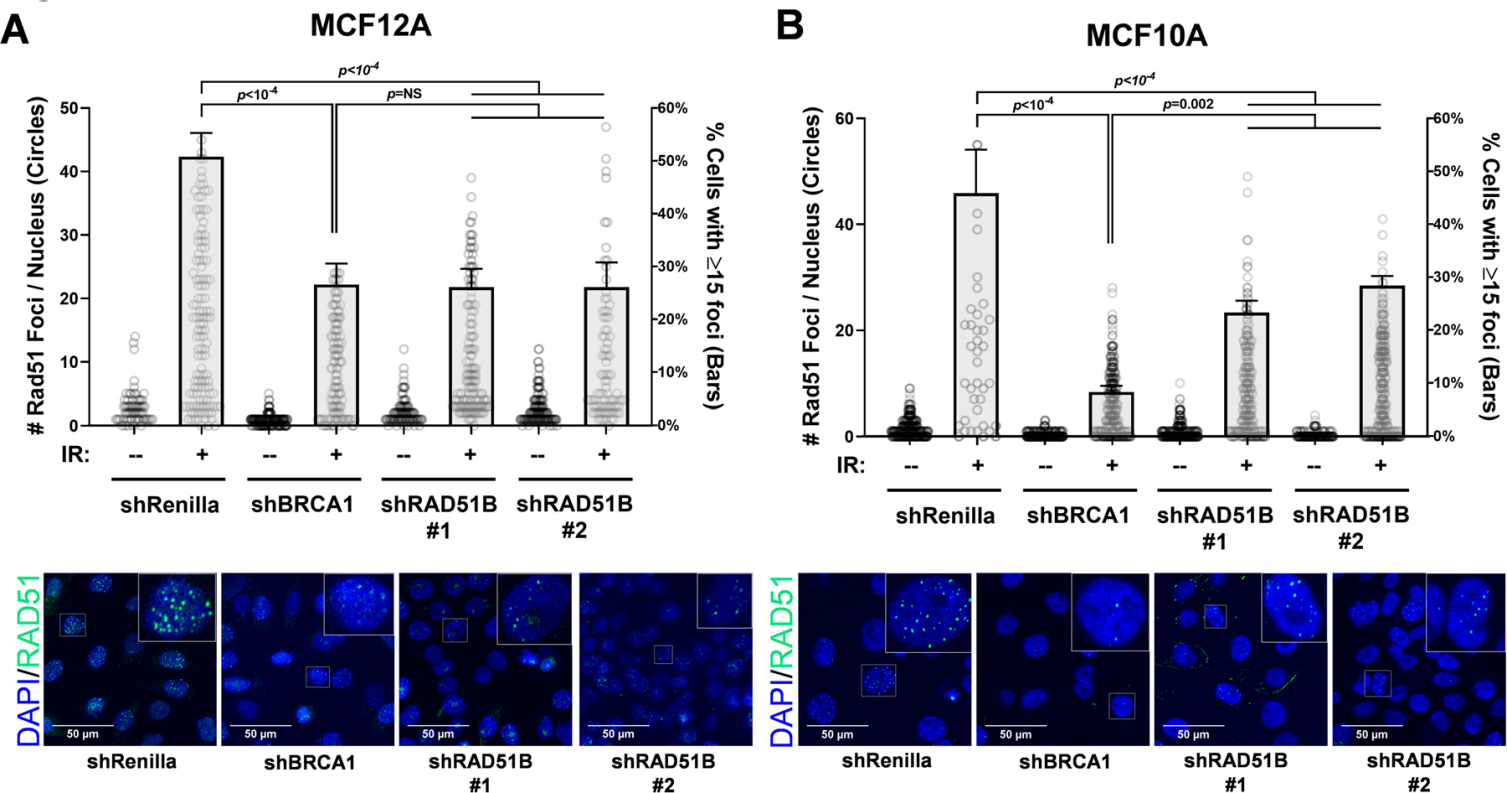
Functional characterization of RAD51B-deficiency on efficiency of homologous recombination. **A**, **B** RAD51 focus formation assay in MCF-12A and MCF-10A mammary epithelial cells expressing one of two independent *RAD51B* shRNAs, Renilla shRNA (nontarget control), or *BRCA1* shRNA (positive control). Cells were plated onto eight-well chamber slides (EMD Millipore) and exposed to 10 Gy of ionizing radiation or mock irradiated (0 Gy). Nuclear RAD51 foci were visualized and quantified in two independent experiments (Supplemental Methods). Error bars represent standard error of mean (SEM).

**Fig. 4:**
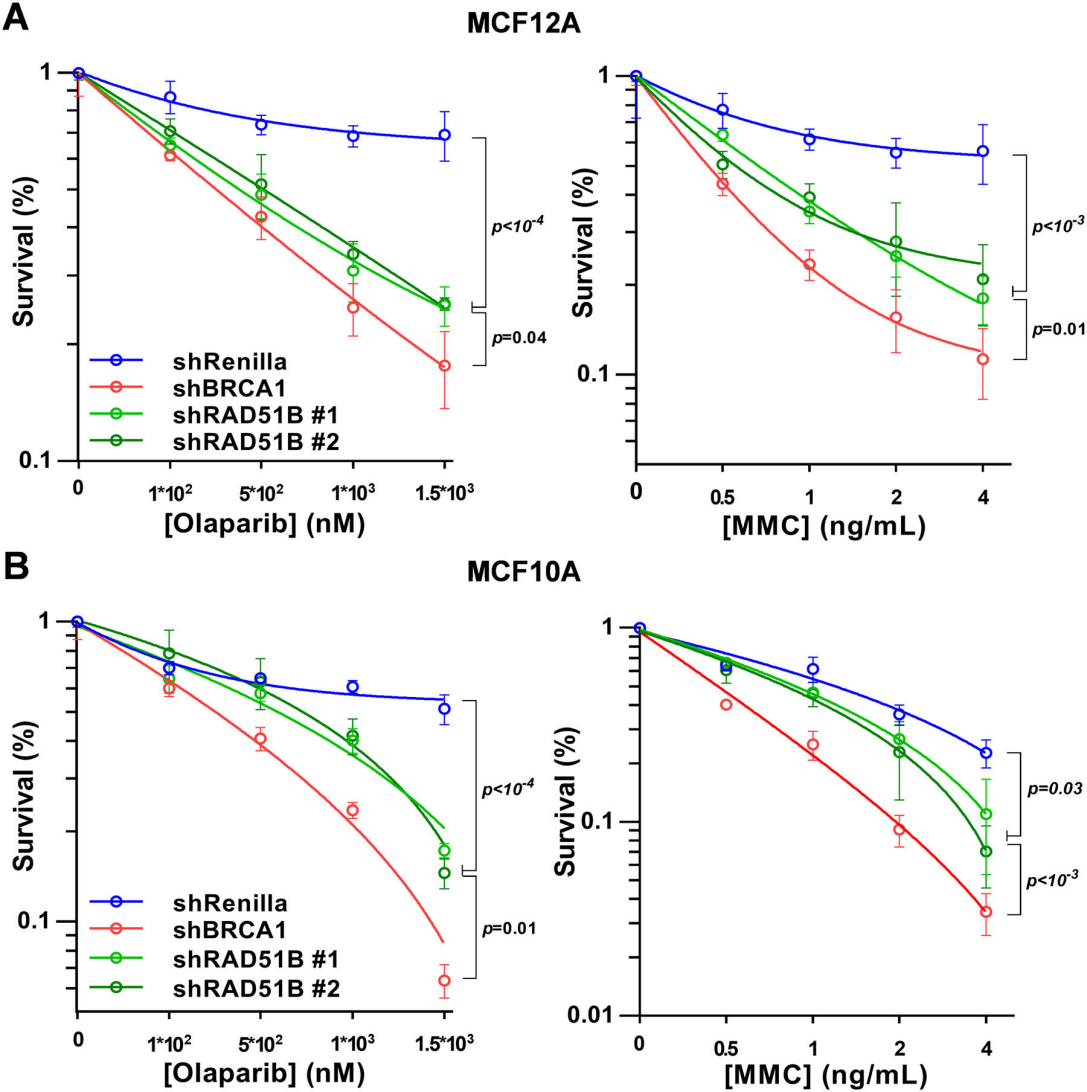
RAD51B-deficiency and sensitivity to MMC and PARPi. **A**, **B** Clonogenic survival assays of the indicated cell lines in response to olaparib or mitomycin C. Cells were expressing one of two independent *RAD51B* shRNAs, Renilla shRNA (nontarget control), or *BRCA1* shRNA (positive control). Error bars represent SEM.

To assess the impact of RAD51B-deficiency on HR efficiency, we quantified the effect of *RAD51B* silencing on the fidelity of HR, as defined by the accumulation of RAD51 nuclear foci following DNA damage, in two breast epithelial cell lines, MCF-10A and MCF-12A. The accumulation of RAD51 nuclear foci in response to DNA damage reflects the fidelity of upstream components of the HR pathway and has been shown to effectively predict clinical responses to HRD-targeting therapies, including PARP inhibition^19,20^. In comparison to a negative control (shRenilla), expression of two different shRNAs against *RAD51B* expression resulted in significantly impaired RAD51 nuclear foci formation (Fig. 3). Silencing of *RAD51B* also resulted in increased sensitivity to olaparib and mitomycin-C, agents that are both known to target HRD cancers (Fig. 4). RAD51B-deficient cells were slightly less sensitive to both agents than BRCA1-deficient cells, concordant with the intermediate LST and signature 3 phenotypes observed for such tumors (Fig. 1B).

As noted in our Methods, loss-of-function *RAD51B* alleles were defined as those harboring a single nucleotide variant (SNV) or insertion or deletion (indel) predicted to result in a frameshifted or truncated reading frame. Nevertheless, several truncating alleles in our study, including the c.139 C > T(p.Arg47*) variant carried by 7/13 patients in our cohort, have not been formally classified as pathogenic or likely pathogenic by American College of the Medical Genetics (ACMG) criteria. To provide supporting evidence that the truncating alleles present in our cohort are deleterious to RAD51B function, we used the standard DR-GFP reporter assay established in U2OS cells, as previously described^21^, to test the HR phenotype of the c.139 C > T(p.Arg47*) variant. As shown in Supplementary Fig. 2, this variant was unable to complement HR proficiency beyond that observed with expression of an empty vector, in contrast to complementation with WT *RAD51B*, which resulted in an approximately twofold increase in recombination repair.

## Discussion

Pathogenic germline mutations in *BRCA1*, *BRCA2*, *PALB2*, *RAD51C*, and *RAD51D*, all of which are involved in the HR pathway, are known to confer high or moderate penetrance susceptibility to ovarian and/or triple-negative breast cancer. In contrast to these known cancer predisposition genes, the role of *RAD51B* in conferring susceptibility to hereditary breast and ovarian cancer has not yet been established. Our findings provide evidence that rare loss-of-function germline variants in *RAD51B* (found in 9/3422 [0.26%] consecutive breast or ovarian cancer cases who consented for germline analysis) are indeed associated with an increased risk of breast and ovarian cancer among women.

Our definition of loss-of-function *RAD51B* alleles included all SNVs and indels predicted to result in a frameshifted or truncated reading frame. Despite encoding for truncated and/or frameshifted RAD51B protein, several *RAD51B* alleles in our cohort could arguably be considered variants of unknown significance by formal ACMG criteria^22^, including the c.139 C > T(p.Arg47*) variant carried by seven patients in our cohort. Our experimental data using the DR-GFP reporter assay of HR proficiency demonstrate that the c.139 C > T(p.Arg47*) variant cannot complement the HR-deficiency observed in RAD51B-deficient cells, and suggests that this allele results in loss of function. While we were unable to test all novel *RAD51B* variants present in the MSK and TCGA cohorts, functional data suggest that the Walker A and B motifs of RAD51B are important for its interaction with RAD51C and for its function in HR^23^. Notably, 11 of the 13 patients in our cohort harbored *RAD51B* alleles predicted to disrupt one or both Walker motifs. As these motifs are important for the interaction of RAD51B with RAD51C and for its function in HR, alleles that truncate or frameshift these domains appear likely to affect protein function. Further experimental data will nevertheless be required to confirm these observations and to assess the pathogenicity of untested alleles that truncate the C-terminus of RAD51B.

In our cohort of 3422 index breast and ovarian cancer cases, we found *RAD51B* loss-of-function germline variants (9/3422 cases) to be almost as common as those affecting *RAD51C* and *RAD51D* (combined 16/3422 cases). The prevalence of mutations affecting these Rad51 paralogs in our cohort was comparable to a prior study of ovarian cancer patients which identified a similar overall prevalence of loss-of-function germline variants in *RAD51B/C/D* (combined 22/3112 cases)^16^. At variance with our data, however, this study identified only 2 patients with alterations in *RAD51B*, with the remaining 20 patients harboring germline inactivation of a *RAD51C* or *RAD51D* allele. This study and others^3,24^, have also demonstrated that carriers of pathogenic *RAD51C*/D mutations tend to develop triple-negative breast cancers, in contrast to the predominantly (4 of 5 cases) hormone receptor-positive breast cancers identified among carriers of *RAD51B* mutations in our cohort. Given the rarity of loss-of-function germline *RAD51B* variants, our study had limited statistical power to assess an association between *RAD51B* inactivation and hormone receptor status among breast cancer cases, and additional cases will be required to confirm the association. One plausible hypothesis for the differences in molecular subtype associated with each mutated paralog relates to the cell of origin and mammary stem/progenitor cell maturation. The observation that *BRCA1* mutation carriers tend to develop triple-negative breast cancers while *BRCA2* mutation carriers show the same range of hormonal receptor subtypes as sporadic breast cancer, has previously been attributed to a role for BRCA1 in the maturation of ER-positive mammary epithelial progenitor cells^25,26^. It remains to be seen, however, whether RAD51C or RAD51D play a similar role in the maturation of mammary progenitor cells.

In addition to providing evidence that *RAD51B* serves as a cancer predisposition gene, our findings confirm that tumors harboring biallelic inactivation of *RAD51B* are deficient in HR and are likely to be sensitive to both PARP inhibitors and interstrand crosslinking agents.

Prior in vitro studies using Chinese hamster cells and the chicken DT40 cell line have demonstrated the classical RAD51 paralogs, including *RAD51B*, to be critical for the integrity of the HR pathway in such model systems^13,27^. Nevertheless, a recent report using human isogenic cell lines suggested that *RAD51B* may be distinct from the other paralogs in terms of its essentiality for HR and cellular viability, with *RAD51B*^*−/−*^ human cells displaying a weaker, intermediate sensitivity to DNA damage^23^, despite being known to function in a heterotypic complex with other paralogs (RAD51C, RAD51D, XRCC2)^13^. Our finding that RAD51B-deficient cells are less sensitive to mitomycin C and olaparib than BRCA1-deficient cells, and yet markedly more sensitive than the corresponding WT controls, are concordant with these reported results. Likewise, our observation that tumors harboring biallelic *RAD51B* inactivation display an intermediate LST and signature 3 features compared to WT and BRCA1/2-deficient tumors, respectively, is suggestive of a significant but moderately attenuated HR-deficient phenotype among such tumors.

Prior genomic data derived from whole genome-sequenced ovarian tumors have suggested that *RAD51B* inactivation may be associated with an enrichment of foldback inversions, a structural variant characterized by inverted duplications^28^. Wang et al. employed hierarchical clustering analysis of 133 whole genome-sequenced ovarian tumors to identify a subgroup characterized by a high prevalence of foldback inversions^28^. Among 24 tumors in this ‘high’ foldback inversion subgroup, seven (29%) were found to harbor *RAD51B* somatic inactivation. This subgroup also exhibited an intermediate elevation of genomic features associated with HRD, and was distinct from a separate HR-deficient subgroup that was enriched with BRCA1- and PALB2-deficient tumors. The nature of our sequencing data (whole exome) unfortunately did not allow us to identify such structural variants, but further examination of this link could help to clarify the genomic consequences of *RAD51B* loss and whether it diverges from other core genes required for HR.

Three male patients in our cohort carrying a *RAD51B* loss-of-function variant were affected by malignant tumors, but none were of mammary origin (Table 2). To our knowledge, there is no published evidence that *RAD51B* loss-of-function variants are associated with cancer predisposition among men. Prior reports have found a common single nucleotide polymorphism (SNP) in intron 7 of RAD51B (rs1314913) to be associated with a low risk of male breast cancer^29,30^. Orr et al. for example, demonstrated rs1314913 to be significantly associated with male, but not female breast cancer risk, with an odds ratio of ~1.6^29^. As non-coding SNPs often influence their target genes through long-range chromosomal interactions^31,32^, rather than the closest or most biologically relevant nearby gene^30,33^, further studies are needed to identify the causative haplotype underlying the association. Given the absence of functional data suggesting a link between the rs1314913 SNP and RAD51B function, as well as the fact that none of the men in our cohort harboring a loss-of-function *RAD51B* allele presented with breast cancer, there appears to be minimal evidence of a cancer predisposition phenotype for loss-of-function *RAD51B* variants among men at this juncture.

Our study has important limitations. The frequency of *RAD51B* loss-of-function variants in our cohort was compared to the entire gnomAD database, hence we cannot exclude the possibility that the proportion of ethnicities do not exactly match the gnomAD database, given that the ethnicities of the individuals in our cohort reflect the diversity of a New York City population. Interestingly, 6/13 of the RAD51B carriers were Hispanic while 2/13 were Turkish. This raises the possibility that RAD51B pathogenic variants may contribute more to breast and ovarian cancer susceptibility in non-Caucasian populations. Further studies are warranted to confirm the role of *RAD51B* in conferring breast and ovarian cancer susceptibility in additional populations and to define the inclusion of *RAD51B* in panels for genetic risk prediction multigene assays. An additional 3 breast and ovarian cancer cases were not analyzed in this cohort because in additional to *RAD51B* germline mutations, they carried another high or moderate penetrance mutation in a cancer susceptibility gene (1 each *BRCA2*, *RAD51D*, *ATM*). However, it is a well-known phenomenon that individuals can have germline mutations in more than one cancer susceptibility gene, including simultaneous *BRCA1* and *BRCA2* mutations^34–36^, and that either one may be an initiating factor in an individual’s cancer or the mutations in the HR pathway may act synergistically in tumor development. Three patients in our MSK cohort harbored germline *RAD51B* variants that affected canonical dinucleotide splicing sites. While computational algorithms predict that all 3 of these variants effect splicing and the positive predictive value of canonical splice site donor or acceptor disruption has previously been shown to approach >95%^37^, RNA sequencing data for these cases was unavailable and we could not directly assess for intron retention/deleterious splicing at the transcript level.

Taken together, the data presented here support the notion that *RAD51B* loss-of-function germline variants result in increased breast and ovarian cancer predisposition and that biallelic loss of *RAD51B* in tumor cells leads to an HRD phenotype and potential sensitivity to HRD-targeting therapies. If the risk estimates reported here are confirmed, our findings indicate that *RAD51B* should be included in multigene panel testing for genetic risk prediction.

## Methods

### Patient data

Individuals with a *RAD51B* loss-of-function germline variant were identified from the cohort of patients who consented to undergo somatic and germline analysis under an Institutional Review Board-approved protocol at Memorial Sloan Kettering Cancer Center (MSKCC) and were profiled using the MSK-IMPACT assay (*n* = 9287). Three breast and ovarian cancer cases were excluded from the analysis, as in addition to harboring a *RAD51B* loss-of-function germline variant, they also carried an additional mutation in a cancer susceptibility gene (1 each *BRCA2*, *RAD51D*, or *ATM* [MIM: 607585]). None of the *RAD51B*-associated cancers included in this analysis harbored germline alterations in known cancer susceptibility genes. All germline *RAD51B* variants were reviewed by a board-certified molecular pathologist (D.M.); loss-of-function alleles were defined as those harboring an SNV or indel resulting in a frameshifted or truncated reading frame, including start/stop codon changes or canonical dinucleotide splice site disruption. Surgical pathology reports and medical records were reviewed (D.M., Y.K). to establish clinico-pathologic features and family histories. For breast cancer cases, estrogen receptor (ER), progesterone receptor (PR), and HER2 status were assessed following the American Society of Clinical Oncology/College of American Pathologists guidelines^38^.

### Statistical analysis

The carrier frequency of loss-of-function variants in gnomAD was compared to the carrier frequency in the cancer patient cohort using the Fisher exact test (RStudio 1.0.143). All statistical tests were two-sided with *p* < 0.05 considered statistically significant.

### Massively parallel sequencing and bioinformatics analysis

Tumor and matched normal DNA samples were subjected to targeted massively parallel sequencing using the Memorial Sloan Kettering Integrated Mutation Profiling of Actionable Cancer Targets (MSK-IMPACT) assay, which targets all exons and selected introns of 410 (*n* = 1) or 468 (*n* = 12) cancer genes, as previously described^39,40^. Sequencing data was analyzed for SNVs and small insertions and deletions (indels) as previously described^41^. Loss-of-function alleles were defined as those harboring an SNV or indel resulting in a frameshifted or truncated reading frame, including start/stop codon changes or canonical dinucleotide splice site disruption. FACETS was used to determine CNAs and whether the WT copy of *RAD51B* was subject to loss of heterozygosity (LOH) in individuals who harbor germline *RAD51B* truncating variants^42^. Mutational hotspots were annotated according to Chang et al.^43^. Data for TCGA cases with *RAD51B* germline variants were downloaded from the National Cancer Institute (NCI) Genomic Data Commons legacy archive. Signature Multivariate Analysis (SigMA), a tool to detect the HRD mutational signature Sig3 from targeted gene panels^44^, was employed for samples with ≥5 somatic SNVs. To assess structural/copy number signatures of HRD, LSTs were defined, and a cut-off ≥15 was employed to define LST-high cases, as previously described^17^. To eliminate the possibility of confounding by treatment-related genomic changes previously treated metastatic samples were excluded from mutational and structural signature analysis. As LOH events themselves are CNAs that could contribute to LST signal, cases harboring *RAD51B* LOH with a loss-of-function allele were compared to TCGA cases harboring *RAD51B* LOH with a WT allele. For this analysis, cases harboring a deleterious HR gene mutation or cancer type not present in our cohort were excluded.

### Cell culture

The immortalized but non-transformed MCF-12A and MCF-10A breast epithelial cell lines were purchased from American Type Culture Collection (ATCC). These cells were short tandem repeat (STR) profiled by the provider and used within the first 20 passages. Cell lines were tested for mycoplasma infection using the Universal Mycoplasma Detection Kit (ATCC) and grown in Dulbecco’s modified Eagle’s medium (DMEM):F12 medium supplemented with 5% horse serum (ThermoFisher), 20 ng/ml epidermal growth factor (PeproTech), 500 ng/ml hydrocortisone (Sigma), 10 µg/ml insulin (Sigma), and 100 ng/ml cholera toxin (EMD Millipore).

### Plasmid construction, lentivirus production, and transduction

Gene knockdowns were achieved using a doxycycline-inducible shRNA strategy. The LT3RENIR lentiviral miR-E-based expression vector backbone was purchased from the MSKCC Gene Editing and Screening Core Facility. For generation of miR-E shRNAs, 97-mer oligonucleotides were purchased (IDT Ultramers) coding for predicted shRNAs using an siRNA prediction tool (Splash RNA, http://splashrna.mskcc.org/). 97-mer oligonucleotides were PCR amplified using the primers miRE-Xho-fw and miRE-Eco-rev. PCR products were purified and both PCR product and LT3RENIR vectors were double digested with EcoRI-HF and XhoHI. PCR product and vector backbone were ligated and transformed in Stbl3 competent cells and grown at 32 °C overnight. Colonies were screened using the miRE-fwd primers. Sequences for 97-mer oligonucleotides and PCR/sequencing primers are listed in the Supplementary Materials. Lentiviral particles expressing shRNA hairpins were generated using Lenti-X 293T cells (Takara). One day prior to transfection, 3.8 million Lenti-X 293T cells were seeded in 10 cm plates. Each 10 cm plate was transfected with a LT3RENIR-based shRNA expression construct along with packaging plasmids psPAX2 and pMD2.G (Cellecta) using polyethylenimine (Sigma). Twenty-four hours after transfection, media containing DNA transfection mixture was replaced with regular DMEM supplemented with 10% FBS. Media containing lentivirus was harvested at 24, 48, and 72 h, prior to being pooled, mixed with Lenti-X concentrator solution, centrifuged, and resuspended in PBS. All shRNAs were assessed at single copy genomic integration by infecting target cell population at <20% of their maximal infection rate, guaranteeing <2% cells with multiple integrations. Transduced cell populations were selected 48 h after infection, using 500–2000 μg/ml G418 (Geneticin, Gibco-Invitrogen).

### Immunoblotting

Whole-cell extracts were prepared by lysing cells in radioimmunoprecipitation buffer. Protein (100 g) was loaded into 3–8% Tris acetate gels (Thermo Fisher), subjected to SDS-PAGE, transferred onto nitrocellulose, and blocked with 5% milk–TBST (for 1 h at room temperature. Immunodetection was performed using the following antibodies: anti-RAD51B (sc-53430; Santa Cruz); anti-BRCA1 (OP-92; Calbiochem); anti-Actin (ab14128; Abcam). All immunoblots were derived from same experiment and processed in parallel.

### RAD51 foci formation assay

Cells were plated onto eight-well chamber slides (EMD Millipore) and exposed to 10 Gy of ionizing radiation or mock irradiated (0 Gy). After 4 h, cells were fixed, permeabilized, and co-immunostained with primary antibodies targeting RAD51 (polyclonal rabbit antibody, PC-130; EMD Millipore). Nuclei were counterstained with 4′,6-diamidino-2- phenylindole (DAPI). Nuclear RAD51 foci were visualized and quantified in a minimum of 200 cells in three independent experiments with a Zeiss LSM 880 confocal microscope.

### Clonogenic survival assays

MCF-12A and MCF-10A cells were seeded in six-well plates and allowed to attach for 4 h before treatment with olaparib (Selleckchem AZD2281) or mitomycin-C (Sigma 10107409001) at the indicated doses. After 10–14 days cells were washed, fixed with methanol, and stained with crystal violet. Colonies containing more than 50 cells were counted. Results were normalized to untreated cells; for each genotype cell viability of untreated cells was defined as 100%.

### Direct repeat-green fluorescent reporter (DR-GFP) assay

To assess the influence of the NM_002877.6[RAD51B]: c.139 C > T(p.Arg47*) variant on RAD51B-dependent HR, we used the standard DR-GFP reporter assay established in U2OS cells, as previously described^21^. In brief, these cells were modified to express an inducible shRNA against the 5′ untranslated region (UTR) of *RAD51B*. Seventy-two hours after introducing doxycycline to induce expression of RAD51B shRNA, cells were transfected with an expression vector encoding WT RAD51B, the R47Ter variant (NM_002877.6[RAD51B]: c.139 C > T[p.Arg47*]), or an empty reading frame (all expression vectors lacked the 5′UTR of *RAD51B*). A second transfection was performed 24 h after the first to introduce vectors encoding the I-SceI endonuclease, GFP (positive transfection control) or empty vector (negative control). Forty-eight hours after the second transfection, cells were trypsinized and single cell suspensions were analyzed by flow cytometry

### Reporting summary

Further information on research design is available in the Nature Research Reporting Summary linked to this article.

## Supplementary information

Supplementary Information

Reporting Summary

## Data Availability

Clinicopathologic data are available in the Supplementary Data. Additional validation for isogenic cell lines are available in the Supplementary Materials. Identifying information for the patients is not available to protect patient privacy. Please note that the materials and reagents used in this study may be subject to an institutional material transfer agreement. All other data generated and analysed during this study are publicly available as described in the following data record: 10.6084/m9.figshare.14665086^45^.
