# Supplementary material for: Germline RAD51B variants confer susceptibility to breast and ovarian cancers deficient in homologous recombination
Source: NPJ Breast Cancer. 2021 Oct 11;7:135. doi: 10.1038/s41523-021-00339-0 (PMC8505423; doi:10.1038/s41523-021-00339-0)
Supplement: Supplementary file 1 — Supplementary Information [file 41523_2021_339_MOESM1_ESM.pdf]

SUPPLEMENTAL TABLE

Supplementary Table 1. Germline *RAD51B* pathogenic variant carriers in TCGA cohort.

ID	TCGA Case ID	Sex	Tumor type	*RAD51B* variant	*RAD51B* mutation type	Mean allele frequency (MAF) in gnomAD	Ethnicity-specific MAF in gnomAD	Age	Hormone receptor status	Ethnicity
TCGA-01	TCGA-A8-A06X[1]	Female	Breast Invasive Ductal Carcinoma	NM_002877.6(RAD51B): c.139C>T(p.Arg47Ter)	Truncating SNV	38/282688 (0.01%)	N/A	77	ER+, PR-, HER2+	N/A
TCGA-02	TCGA-B5-A1N2[2]	Female	Uterine Endometrioid Carcinoma	c.22C>T(p.Arg8Ter)	Truncating SNV	Absent	Absent	70	NP	N/A
TCGA-03	TCGA-E2-A573[3]	Female	Breast Invasive Ductal Carcinoma	c.22C>T(p.Arg8Ter)	Truncating SNV	Absent	Absent	48	ER-, PR-, HER2-	Caucasian
TCGA-04	TCGA-42-2588[4]	Female	High-Grade Serous Ovarian Cancer	c.414_418delGGTGT(p.Val139HisfsTer2)	Truncating Indel	Absent	Absent	61	NP	
TCGA-05	TCGA-DD-A3A5[5]	Female	Liver Hepatocellular Carcinoma	NM_002877.6(RAD51B): c.139C>T(p.Arg47Ter)	Truncating SNV	38/282688 (0.01%)		66	NP	Caucasian
TCGA-06	TCGA-CD-A4MI[6]	Male	Stomach Adenocarcinoma	c.592G>T(p.Glu198Ter)	Truncating SNV	Absent	Absent	62	NP	Caucasian

Abbreviations: TCGA, The Cancer Genome Atlas; gnomAD, The Genome Aggregation Database; ER, estrogen receptor; PR, progesterone receptor; HER2, human epidermal growth factor receptor 2; NP, not performed.

Supplementary Table 2. Sequences for 97-mer oligonucleotides and PCR/sequencing primers used for cloning of miR-E shRNAs:

Oligonucleotide	Sequence
shRenilla	TGCTGTTGACAGTGAGCGCAGGAATTATAATGCTTATCTATAGTGAAGCCACAGATGTATAG ATAAGCATTATAATTCCTATGCCTACTGCCTCGGA
shRAD51B #1	TGCTGTTGACAGTGAGCGAACCTGTGATGAAGTTCTACAATAGTGAAGCCACAGATGTATTGTA GAACTTCATCACAGGTGTGCCTACTGCCTCGGA
shRAD51B #2	TGCTGTTGACAGTGAGCGCCCCGGCATGGGTAGCAAGAAATAGTGAAGCCACAGATGTATTTCTTGCTACCCATGCCGGGTTGCCTACTGCCTCGGA
shRAD51B #3	TGCTGTTGACAGTGAGCGCCCCGGCATGGGTAGCAAGAAATAGTGAAGCCACAGATGTATTTCTTGCTACCCATGCCGGGTTGCCTACTGCCTCGGA
shBRCA1	TGCTGTTGACAGTGAGCGCTAGCTGGTTTCCCTAAGTTTATAGTGAAGCCACAGATGTA TAAACTTAGGGAAACCAGCTATTGCCTACTGCCTCGGA
miRE-Xho-fw	TGAACTCGAGAAGGTATATTGCTGTTGACAGTGAGCG
miRE-Eco-rev	TGAACTCGAGAAGGTATATTGCTGTTGACAGTGAGCG
miRE-fwd	TGTTTGAATGAGGCTTCAGTAC

SUPPLEMENTAL FIGURES

Supplementary Figure 1

Large-scale transition (LST) scores in biallelic RAD51B-associated cancers, monoallelic RAD51B-associated cancers, biallelic BRCA1-associated cancers, biallelic BRCA2-associated cancers, and RAD51B wild-type cases with LOH. As LOH events themselves are copy number alterations that could contribute to LST signal, cases harboring RAD51B LOH with a loss-of-function allele were compared to TCGA cases harboring RAD51B LOH with a wild type allele (Mann-Whitney U).

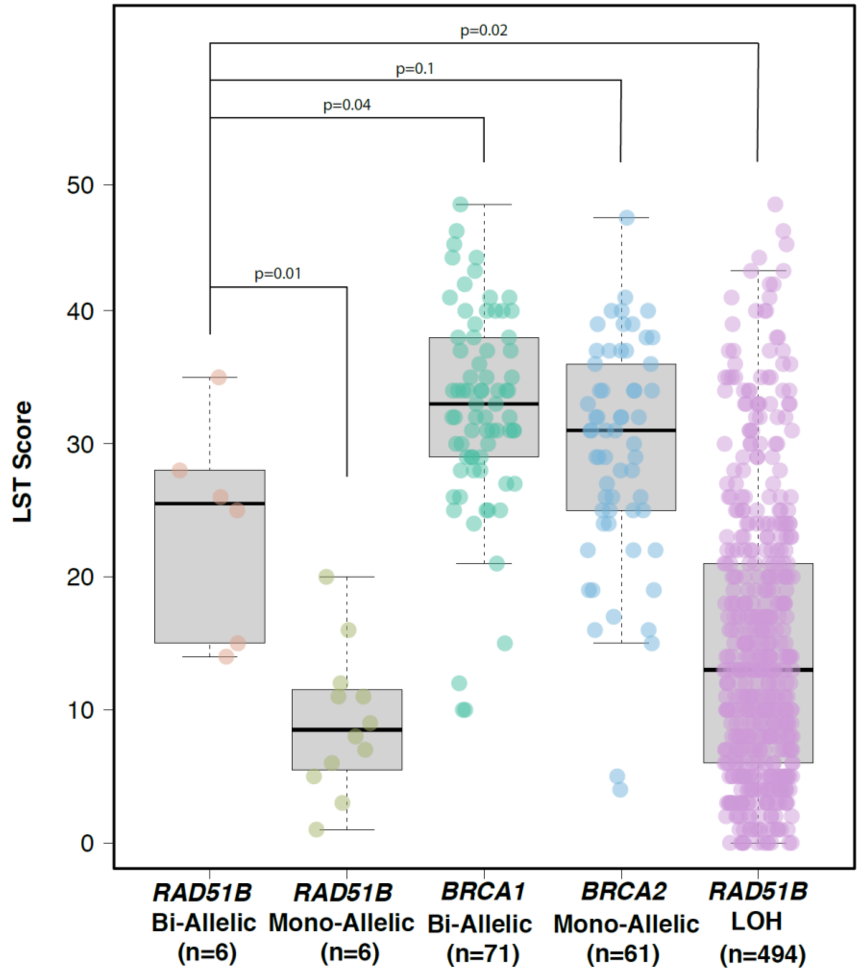

Supplementary Figure 2. Homologous recombination phenotype of *RAD51B* c.139C>T(p.Arg47*) variant.

A) The standard DR-GFP reporter assay established in U2OS cells was used to assess the influence of the NM_002877.6[RAD51B]: c.139C>T(p.Arg47*) variant on RAD51B-dependent homologous recombination. The c.139C>T(p.Arg47*) variant was unable to complement HR proficiency beyond that observed with expression of an empty vector, in contrast to complementation with wild-type RAD51B, which resulted in an approximately two-fold increase in recombination repair. Error bars represent standard error of mean. Student's t-test was used to compare mean GFP% between conditions. B) Corresponding immunoblot of RAD51B expression in U2OS cells expressing an inducible shRNA against the 5' UTR of RAD51B, and transfected with expression vectors encoding wild-type RAD51B, p.Arg47* (NM_002877.6[RAD51B]: c.139C>T[p.Arg47*]), or an empty reading frame (EV). Immunoblotting was performed using a monoclonal antibody (Novus NB100-176) raised against full-length RAD51B (unknown epitope) which may be unable to detect the truncated p.Arg47* variant. Abbreviations: WT, wild type; EV, empty vector, GFP, green fluorescent protein.

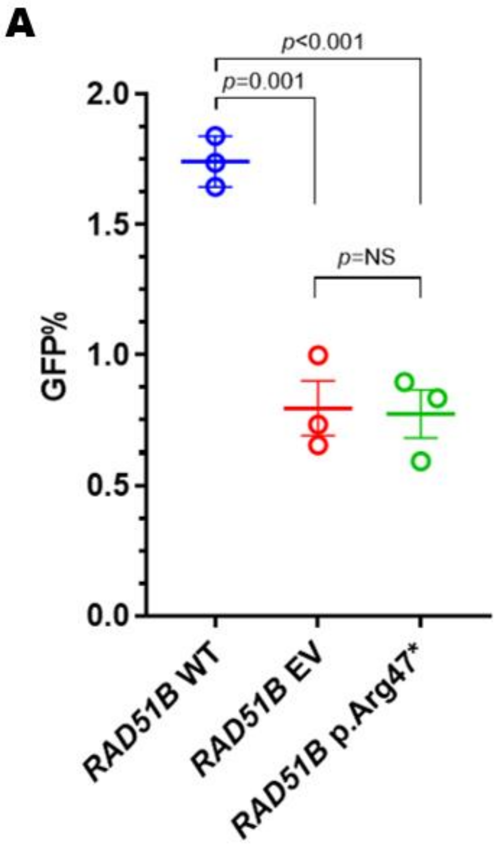

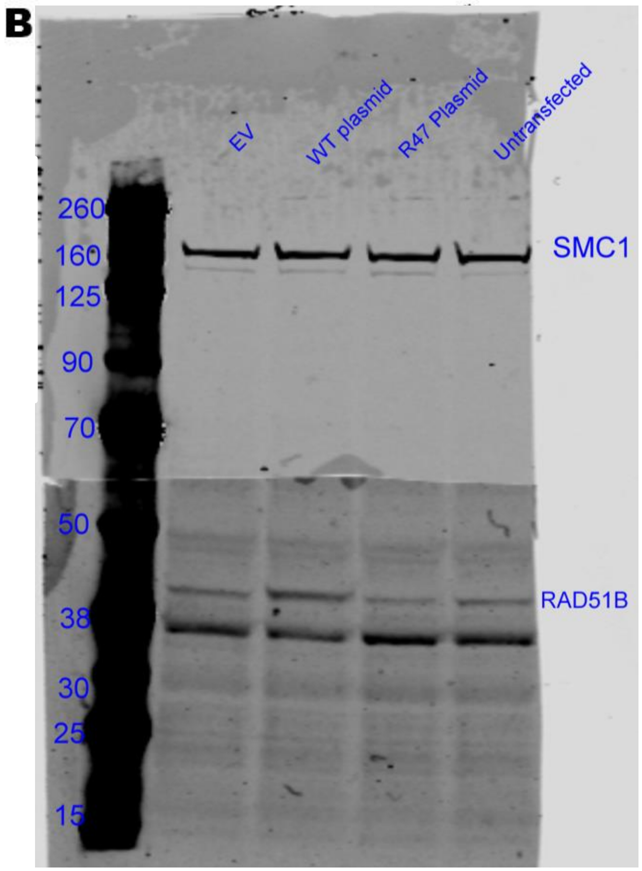

Supplementary Figure 3. Pedigree charts for patients harboring pathogenic germline *RAD51B* variants

MSK_01

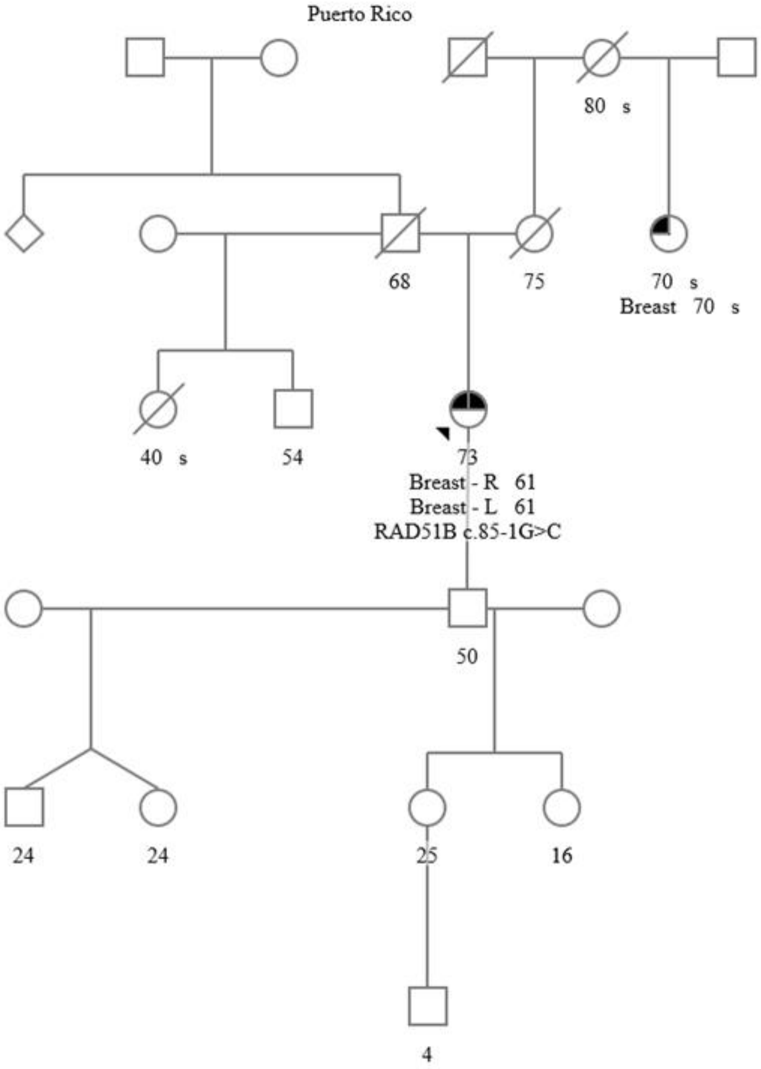

MSK_02

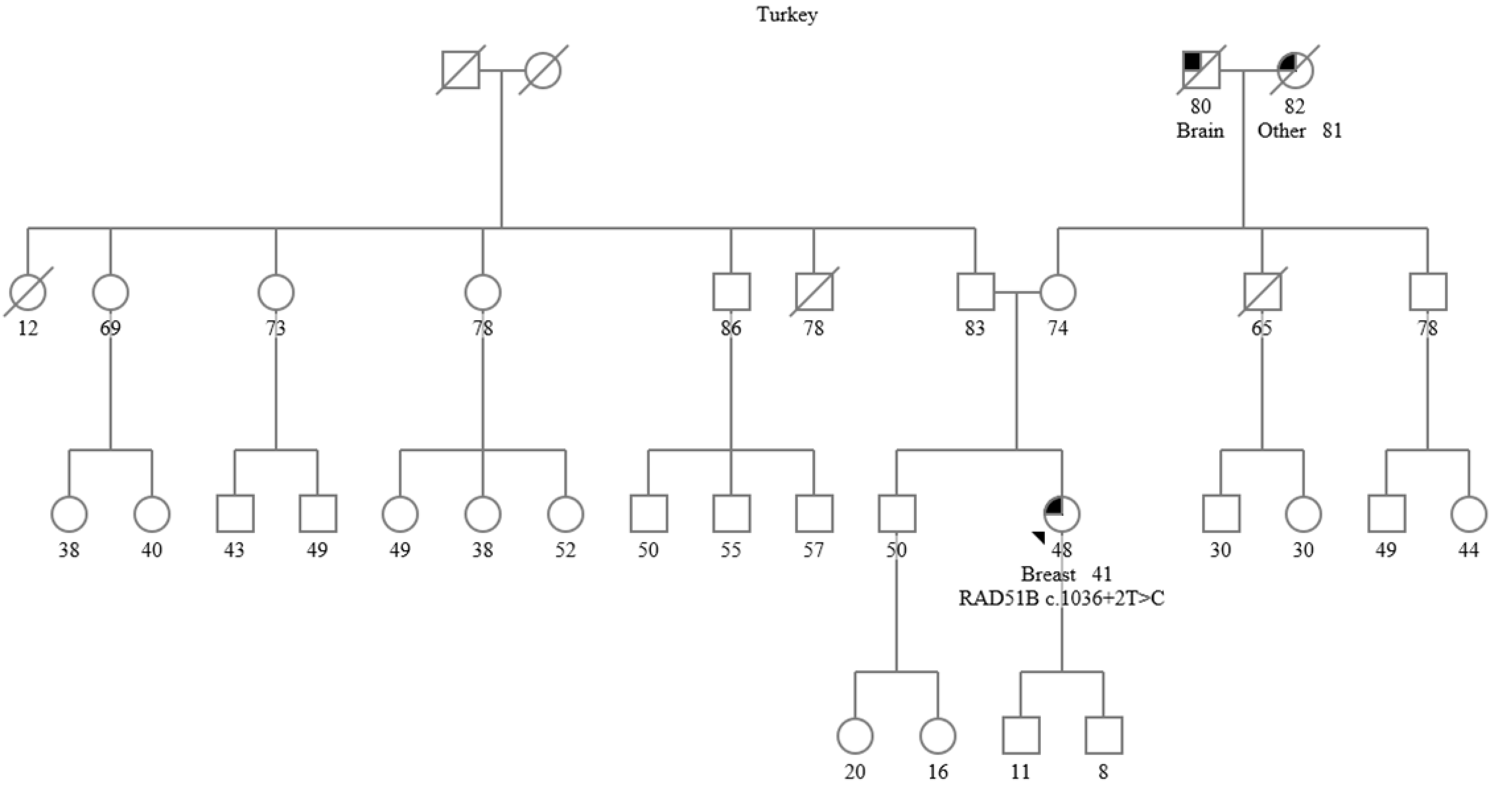

Turkey
80
Brain
82
Other 81
12
69
73
78
86
78
83
74
65
78
38
40
43
49
49
38
52
50
55
57
50
48
Breast 41
RAD51B c.1036+2T>C
30
30
49
44
20
16
11
8

MSK_03

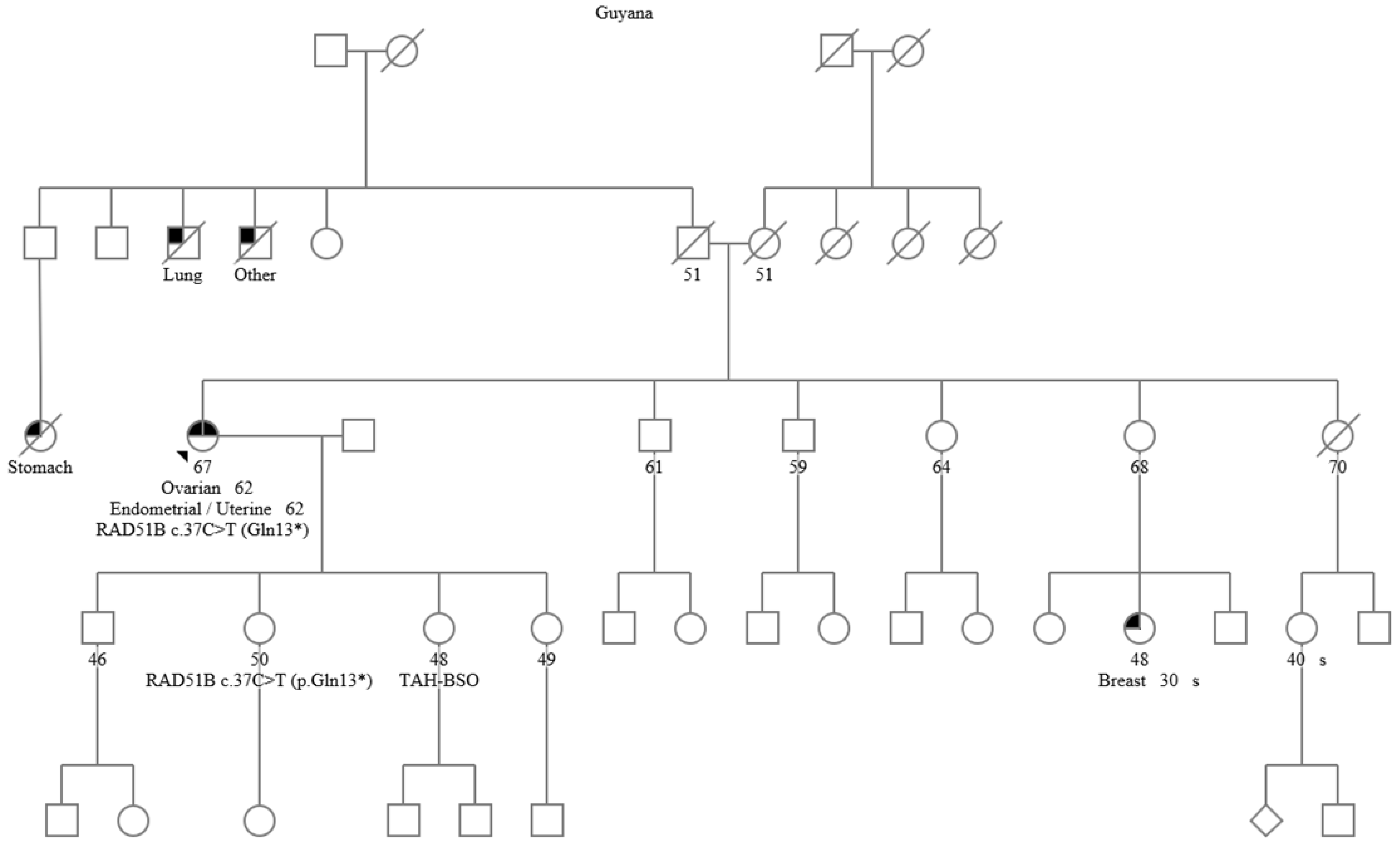
Guyana
Lung
Other
51
51
Stomach
67
Ovarian 62
Endometrial / Uterine 62
RAD51B c.37C>T (Gln13*)
61
59
64
68
70
46
50
RAD51B c.37C>T (p.Gln13*)
48
TAH-BSO
49
48
Breast 30 s
40 s

MSK_04

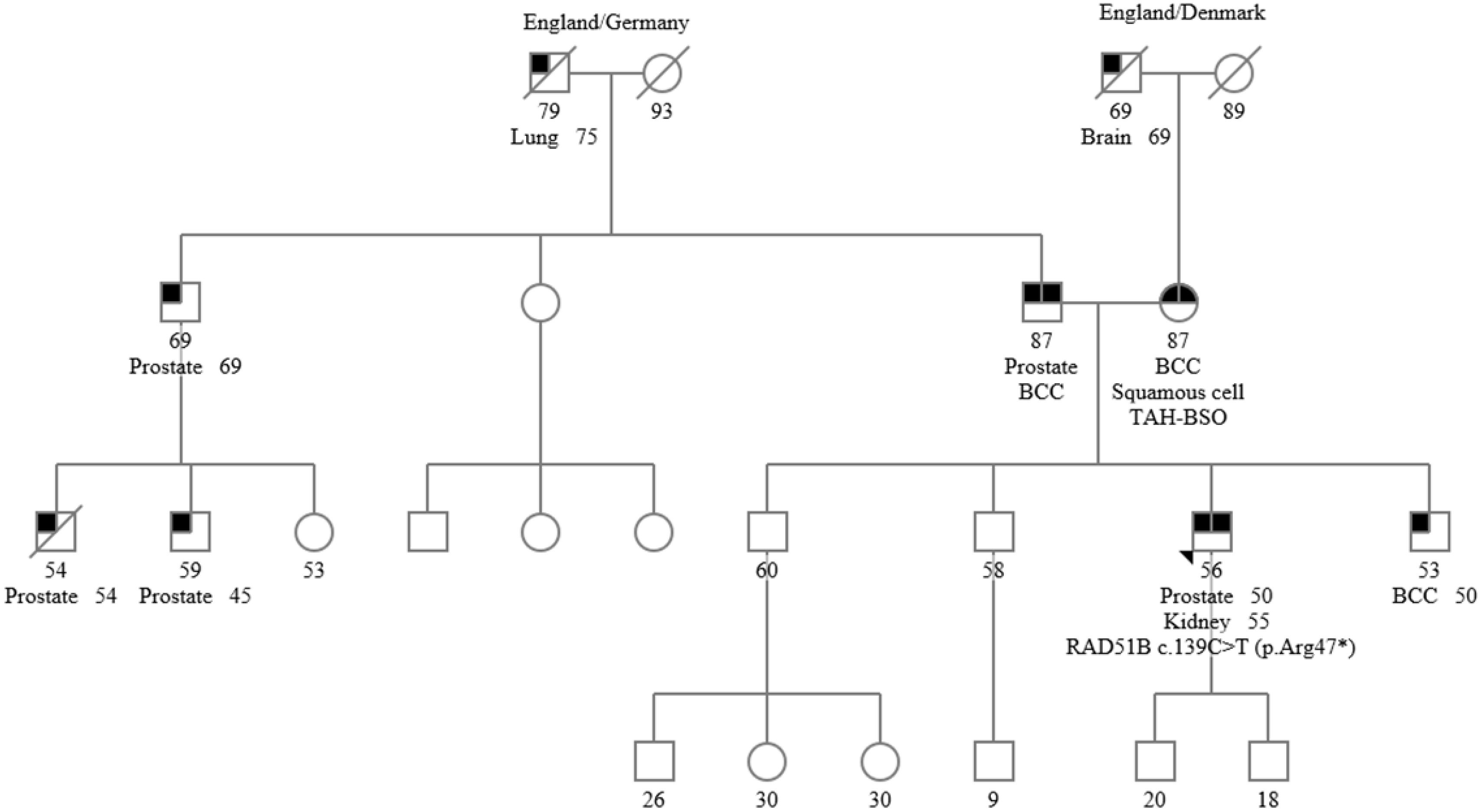

England/Germany
79
Lung 75
93
England/Denmark
69
Brain 69
89
69
Prostate 69
87
Prostate
BCC
87
BCC
Squamous cell
TAH-BSO
54
Prostate 54
59
Prostate 45
53
60
58
56
Prostate 50
Kidney 55
RAD51B c.139C>T (p.Arg47*)
53
BCC 50
26
30
30
9
20
18

MSK_05

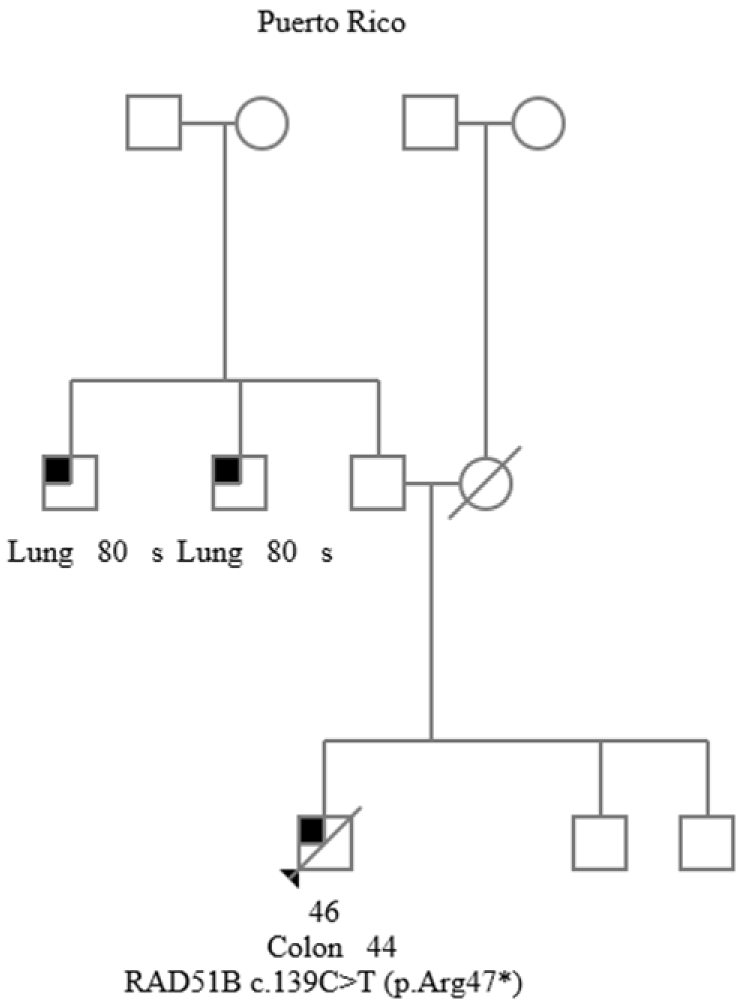
Puerto Rico
Lung 80 s
Lung 80 s
46
Colon 44
RAD51B c.139C>T (p.Arg47*)

MSK_06

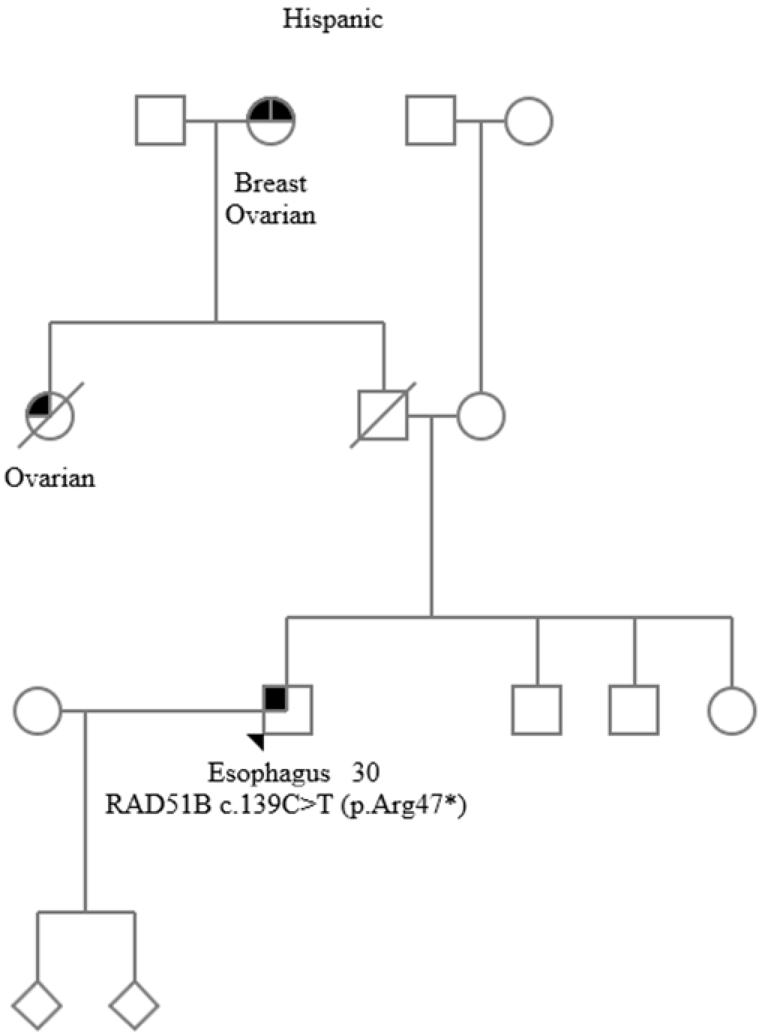
Hispanic
Breast
Ovarian
Ovarian
Esophagus 30
RAD51B c.139C>T (p.Arg47*)

MSK_07

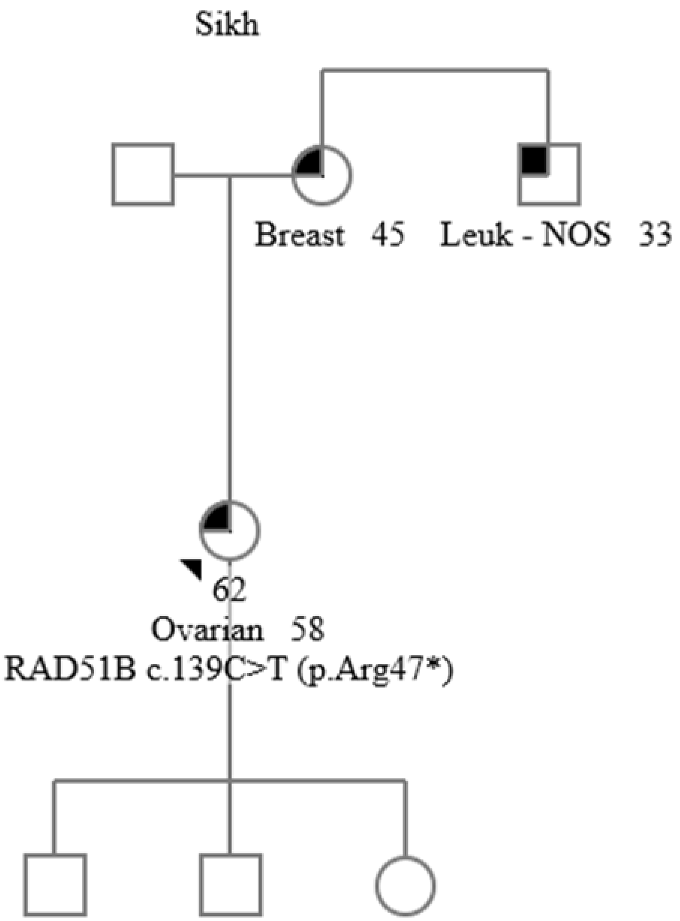
Sikh
Breast 45
Leuk - NOS 33
62
Ovarian 58
RAD51B c.139C>T (p.Arg47*)

MSK_08

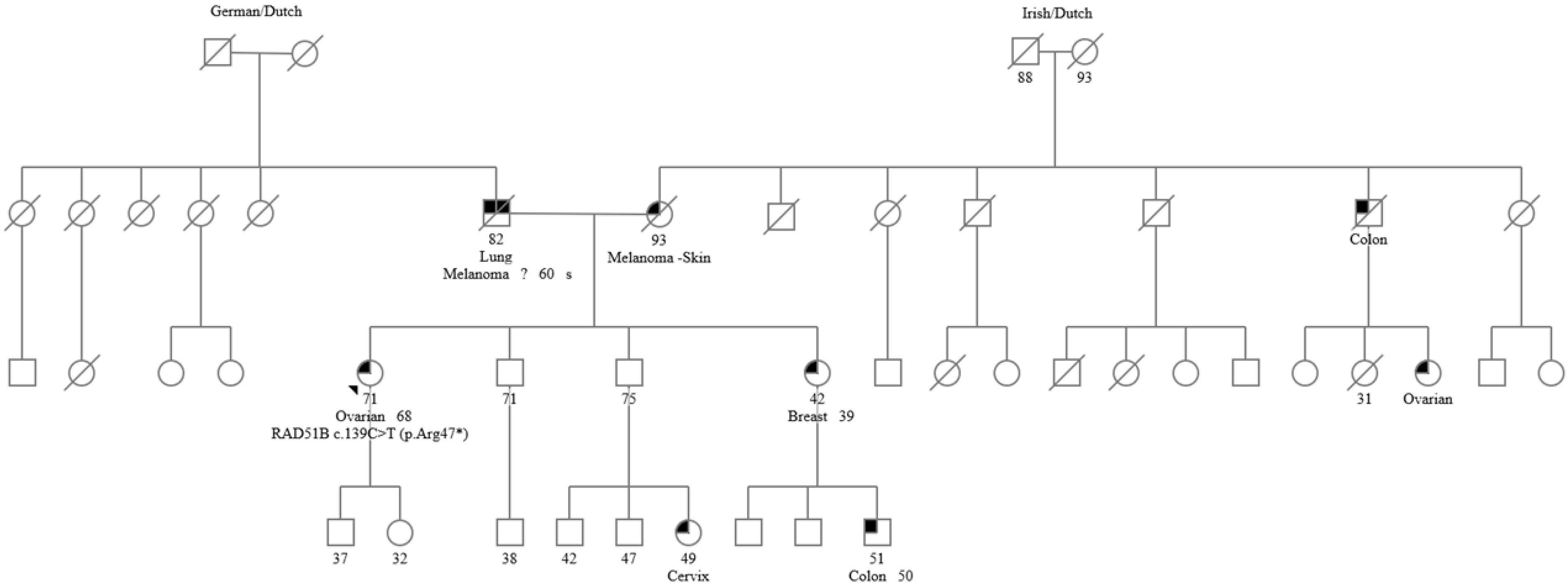
German/Dutch
Irish/Dutch
88
93
82
Lung
Melanoma ? 60 s
93
Melanoma -Skin
Colon
71
Ovarian 68
RAD51B c.139C>T (p.Arg47*)
71
75
42
Breast 39
31
Ovarian
37
32
38
42
47
49
Cervix
51
Colon 50

MSK_09

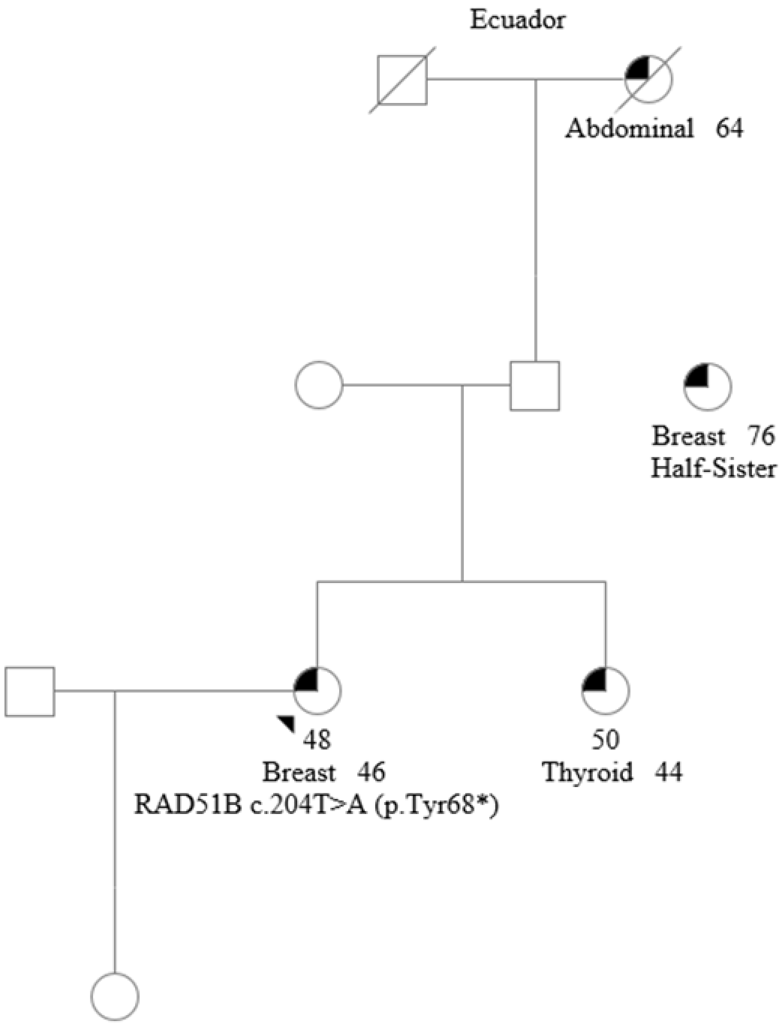

Ecuador
Abdominal 64
Breast 76
Half-Sister
48
Breast 46
RAD51B c.204T>A (p.Tyr68*)
50
Thyroid 44

MSK_10

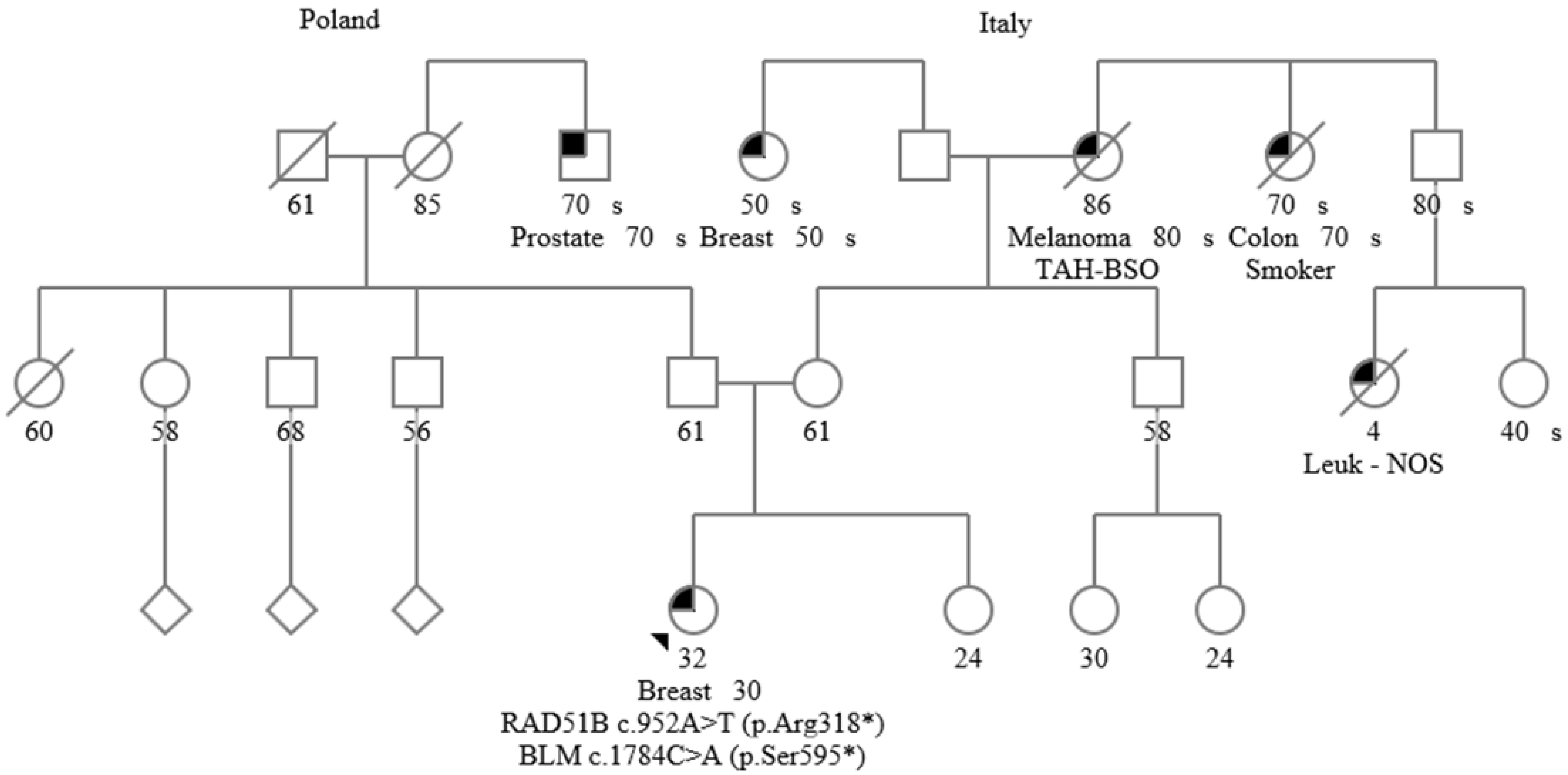
Poland
Italy
61
85
70 s
Prostate 70 s
50 s
Breast 50 s
86
Melanoma 80 s
TAH-BSO
70 s
Colon 70 s
Smoker
80 s
60
58
68
56
61
61
58
4
Leuk - NOS
40 s
32
Breast 30
RAD51B c.952A>T (p.Arg318*)
BLM c.1784C>A (p.Ser595*)
24
30
24

MSK_11

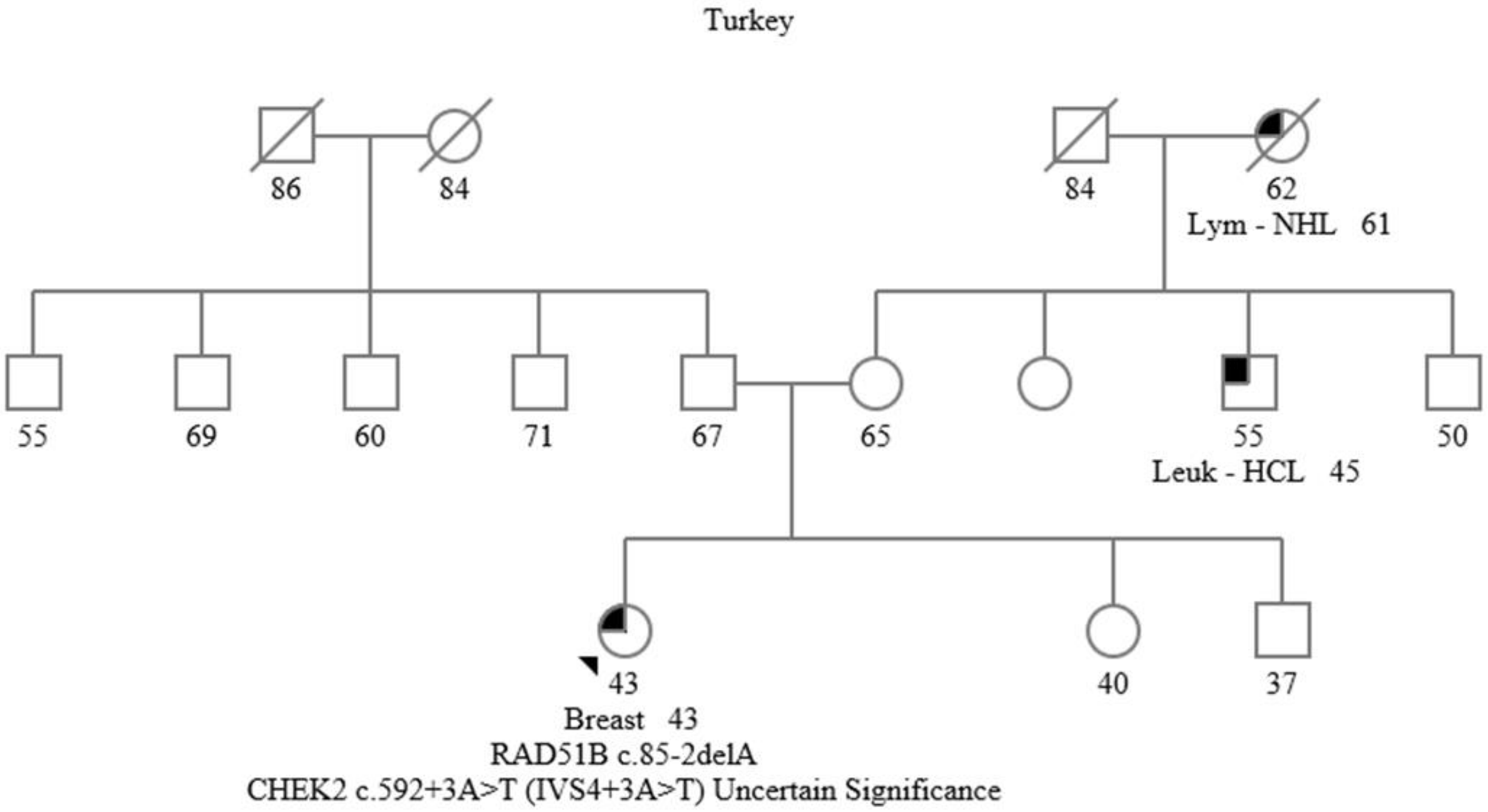

Turkey
86
84
84
62
Lym - NHL 61
55
69
60
71
67
65
55
Leuk - HCL 45
50
43
Breast 43
RAD51B c.85-2delA
CHEK2 c.592+3A>T (IVS4+3A>T) Uncertain Significance
40
37

SUPPLEMENTAL REFERENCES

1. TCGA-A8-A06X *NCI Genomic Data Commons Data Portal* https://identifiers.org/gdc:70931617-b3df-4a12-8e3f-2b2307602f48
2. TCGA-B5-A1N2 *NCI Genomic Data Commons Data Portal* https://identifiers.org/gdc:d79e692c-5053-4484-a180-01a094c5ff45
3. TCGA-E2-A573 *NCI Genomic Data Commons Data Portal* https://identifiers.org/gdc:6429c443-8ac3-407f-bb9c-66420b904bbf
4. TCGA-42-2588 *NCI Genomic Data Commons Data Portal* https://identifiers.org/gdc:654d02ab-05b4-4863-99d9-04d087ff91b4
5. TCGA-DD-A3A5 *NCI Genomic Data Commons Data Portal* https://identifiers.org/gdc:d8549d23-37d4-42bd-b472-f49bbebd09b0
6. TCGA-CD-A4MI *NCI Genomic Data Commons Data Portal* https://identifiers.org/gdc:cda6d95c-be6a-485e-ba9a-c57f3eb3f99a
